# ProCeSa: Contrast-Enhanced Structure-Aware Network for Thermostability Prediction with Protein Language Models

**DOI:** 10.1021/acs.jcim.4c01752

**Published:** 2025-02-24

**Authors:** Feixiang Zhou, Shuo Zhang, Huifeng Zhang, Jian K. Liu

**Affiliations:** † Readline Intelligence, Birmingham B29 6SQ, U.K.; ‡ School of Computer Science, University of Birmingham, Birmingham B15 2TT, U.K.

## Abstract

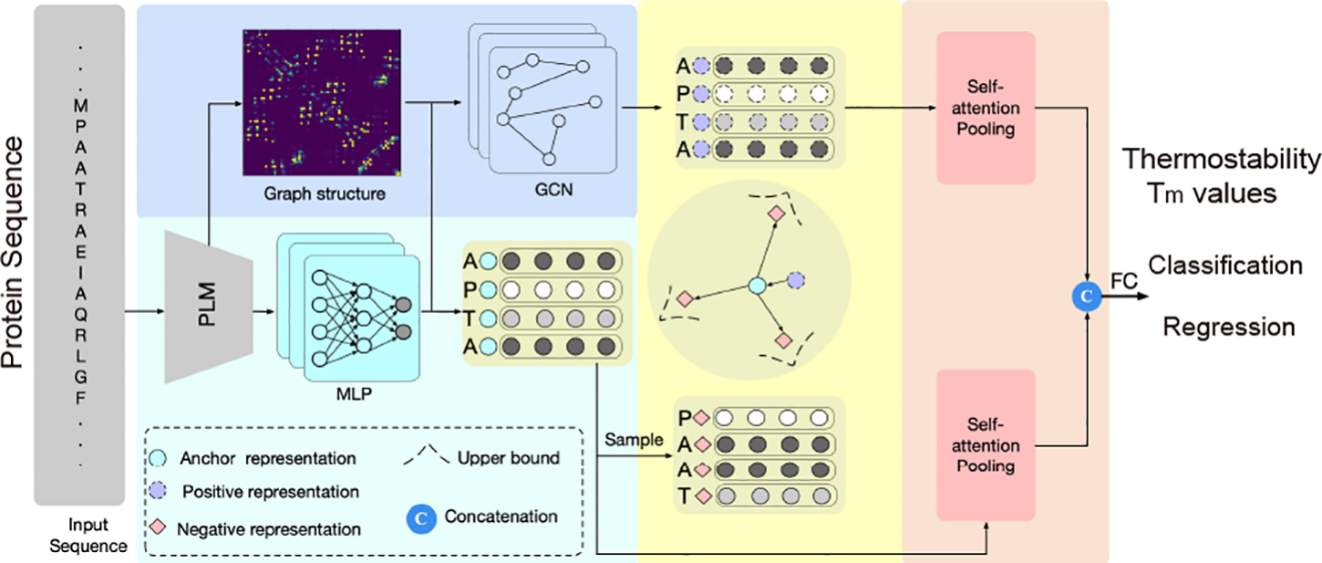

Proteins play a fundamental role in biology, and their thermostability is essential for their proper functionality. The precise measurement of thermostability is crucial, traditionally relying on resource-intensive experiments. Recent advances in deep learning, particularly in protein language models (PLMs), have significantly accelerated the progress in protein thermostability prediction. These models utilize various biological characteristics or deep representations generated by PLMs to represent the protein sequences. However, effectively incorporating structural information, based on the PLM embeddings, while not considering atomic protein structures, remains an open and formidable challenge. Here, we propose a novel Protein Contrast-enhanced Structure-Aware (ProCeSa) model that seamlessly integrates both sequence and structural information extracted from PLMs to enhance thermostability prediction. Our model employs a contrastive learning scheme guided by the categories of amino acid residues, allowing it to discern intricate patterns within protein sequences. Rigorous experiments conducted on publicly available data sets establish the superiority of our method over state-of-the-art approaches, excelling in both classification and regression tasks. Our results demonstrate that ProCeSa addresses the complex challenge of predicting protein thermostability by utilizing PLM-derived sequence embeddings, without requiring access to atomic structural data.

## 1 Introduction

Protein stability is a fundamental aspect that affects the structure, function, and activity of proteins, playing a crucial role in various biological processes.^1^ The ability of proteins to maintain their native conformation and activity under high-temperature conditions is of great importance in various fields, including biotechnology,^2^ enzyme engineering,^3^ and drug design.^4^ Thermostable proteins have attracted significant attention due to their potential applications in industrial production and as biocatalysts for high-temperature reactions. Consequently, the accurate prediction of protein thermostability has emerged as a crucial effort to leverage the full potential of proteins in various biotechnological applications.

The stability of proteins is influenced by a multitude of factors, including intrinsic and extrinsic components. Intrinsic factors involve biological characteristics, such as amino acid distribution,^5^ dipeptide composition,^6,7^ salt bridges,^8,9^ hydrogen bonding,^10^ and hydrophobic interactions,^11^ all of which contribute to the overall stability of the three-dimensional structures of proteins. Additionally, extrinsic factors such as pH, ionic strength, and temperature play a crucial role in modulating protein stability.^12,13^ Among these factors, temperature holds particular significance because of its direct impact on the conformational dynamics of proteins, making it a key determinant of protein thermostability.

The protein thermostability can be measured by biological experiments, which are expensive, time-consuming, and labor-intensive.^14^ However, in recent years, computational methods^12,15−17^ have gained prominence in the prediction of protein thermostability with higher throughput and lower resource costs compared to traditional experimental approaches. Most current state-of-the-art computational approaches rely on different types of features, such as physicochemical properties,^17,18^ amino acid categories,^17^ and sequence-based biological features,^16,19^ which can then be fed into traditional machine learning models or deep learning networks to predict protein thermostability. The amino acid composition and the dipeptide composition were adopted as inputs to the support vector machine (SVM) to predict protein classes (thermophilic or mesophilic) in a small data set.^20^ By combining the biological characteristics of seven groups of protein sequences, a multilayer perceptron (MLP) was used to distinguish thermophilic proteins from mesophilic proteins.^19^ In addition to these approaches, which focus on the classification of the thermostability of proteins, recent work has emerged on thermostability regression. ProTstab2 used gradient boosting to predict protein melting temperatures.^21^ Similarly, DeepET was used to estimate optimal growth temperatures of source organisms using a large data set of 3 million enzymes in a wide range of organisms, where biological characteristics were extracted as input to the model.^16^ Although these methods have achieved promising results in thermostability prediction, they mainly rely on customized biological or physicochemical features, where some irrelevant and redundant features would affect the prediction performance.

Traditional approaches to protein thermostability prediction rely heavily on detailed structural information, particularly when calculating the relative stability changes (ΔΔ*G*) between wild-type and mutated states.^22,23^ These methods require atomic-level structural data to evaluate the conformational energy differences. However, a fundamental challenge exists: while protein sequence databases are vast, high-quality structural data remain scarce for most proteins. Recent breakthroughs in protein structure prediction, particularly AlphaFold2,^24^ have inspired a new approach to address this limitation. Since these models have been trained on large-scale data sets to extract structural information from sequences, researchers have attempted to leverage the powerful feature extraction capabilities. By using the high-dimensional extracted features as input to downstream neural networks, they aim to predict protein thermostability.^25,26^ However, studies have shown that thermostability predictions are highly sensitive to structural accuracy, and even cutting-edge structure prediction models cannot consistently provide the precision needed for reliable thermostability prediction.^27^ Therefore, it is necessary to develop models that directly predict the melting temperature (*T*_m_) from sequence information without relying on detailed structural features. Such models are highly demanding for facilitating protein engineering without expensive wet-lab experiments.^28^

With advances in protein language models (PLMs) that were trained on hundreds of millions of natural protein sequences,^29−32^ transfer learning has been used to extract protein representations generated by language model encoders. Such informative representations have already been shown to be suitable inputs for various predictive tasks.^33,34^ A large-scale protein data set HotProtein includes temperature annotations related to thermostability was proposed.^15^ Alongside this, a novel learning model was introduced, together with the HotProtein data set. By combining the ESM-1B^29^ with the proposed feature augmentation and pretraining schemes, this model improves the prediction performance. However, the pretraining approach can be complex because it requires protein language model ESM-1B and ESM-IF1^35^ simultaneously to process sequence and 3D coordinate information, respectively. Additionally, the existing methods based on PLMs have limited ability to capture both sequence and spatial structure information on protein sequences.

**Figure 1:**
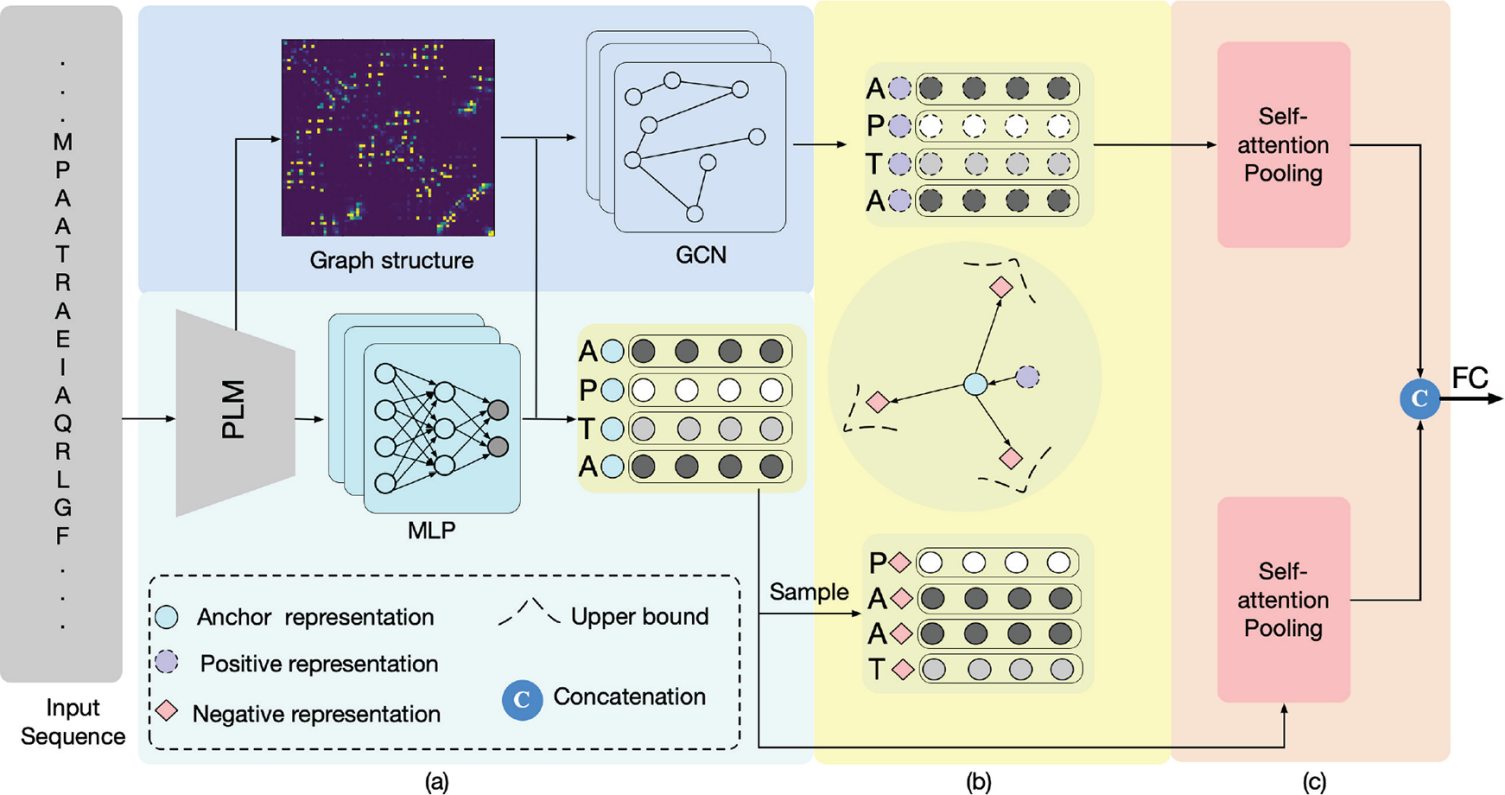
Overview of the proposed ProCeSa for protein thermostability prediction. (a) Sequence and Structure information encoding. A pretrained PLM is first employed to extract sequence features and generate a contact map, representing the graph structure of the protein sequence. These sequence features are further processed through an MLP to produce high-level sequence representations, which are then integrated with the graph structure using a GCN to effectively capture the intrinsic structural relationships among amino acid residues. (b) Contrast-enhanced representation learning. Building on the structure and sequence representations, a contrastive learning scheme is developed, guided by amino acid residue types, to enhance the overall representation quality. (c) Structure-sequence feature aggregation. The enhanced representations are then aggregated using self-attention pooling and subsequently fused to predict protein thermostability.

In this paper, we propose a novel protein contrast-enhanced structure-aware (ProCeSa) model for thermostability prediction from the generated sequence representations and contact maps by PLMs. The contact maps represent pairwise interactions or contacts between amino acid residues within a protein’s 3D structure. Specifically, we first leverage an MLP to extract high-level sequence information based on pretrained sequence representations, followed by designing a graph convolutional network (GCN)^36^ to learn local and global structural features. Afterward, we propose a contrastive learning scheme to enhance the learned sequence and structure representations by discriminating the similarity and dissimilarity between different amino acid residues. As shown in Figure 1, we consider the structure and sequence representations as positive and anchor representations, respectively, followed by constructing the negative representation by sampling the specific amino acid representation from the anchor representation. For positive pairs, we sample the corresponding amino acids from both sequence representation and structure representation, enabling the model to learn the inherent relationships between sequence and structural features that influence protein stability. For negative pairs, we strategically sample amino acids with labels different from the sequence representation, which helps the model distinguish between sequence characteristics associated with varying degrees of thermostability. This sampling and learning process enhances the model’s ability to capture thermostability-related features through joint sequence-structure encoding. The enhanced sequence and structure representations are then aggregated by self-attention pooling to generate feature embeddings associated with protein thermostability.

## 2 Proposed Method

Given a protein sequence consisting of *L* amino acids, its spatial structure can be represented as a graph .  is the set of all nodes in the protein sequence,  represents the edge set  that describes the relationship between any pair of amino acids (*v*_*n*_, *v*_*m*_).  is a set of node features, which is represented as a matrix **X** ∈ .  of the protein sequence can be formulated as an adjacency matrix **A** ∈  where the element *a*_*n*,*m*_ reflects the correlation strength between *v*_*n*_ and *v*_*m*_. Here, we explore two tasks related to protein thermostability prediction, i.e., thermostability classification and regression. For the classification task, each graph (Protein sequence) is associated with a class label *y* ∈ {1, ···, and *C*} where *C* denotes the number of classes. For the regression task, each graph has a ground-truth temperature value (e.g., growth temperature and melting temperature). As shown in Figure 1, our proposed ProCeSa jointly utilizes sequence and structure information to extract rich representations related to thermostability and then incorporates a novel contrastive learning scheme into our model to learn more discriminative representations.

### 2.1 Sequence and Structure Information Encoding

Similar to most existing work,^37,34^ we adopt an MLP to extract a high-level representation with sequence information from per-trained PLMs. Formally, given the pretrained protein feature **P** ∈ , sequence representation **X** ∈  can be formulated as
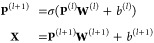
1where **P**^(*l*)^ ∈  denotes the input hidden representation from the previous layer (**P**^(0)^ = **P**) and **W**^(*l*)^ ∈  and *b*^(*l*)^ are trainable parameters. σ is an activation function, such as ReLU(·). *F* is the dimension of pretrained features. The MLP maps the feature dimensions from *F* to *D*, where *D* is the dimension of the learned sequence representations.

To encode structure information within the protein sequence, we apply the widely used GCN^36^ to model structural relations among amino acids, which can be defined as
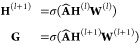
2where **H**^^(0)^^ = **X** and **G** ∈  is the structure representation. **Â** = **D**^–1/2^**ÃD**^–1/2^ ∈  represents a symmetrically normalized adjacency matrix. **D** ∈  is the diagonal matrix, where **D**_*ii*_ = ∑_*j*_(**Ã**_*i j*_) + *c*, and *c* is a small constant avoiding empty rows. **Ã** = **A** + **I**_*L*_ where **I**_*L*_ is the identity matrix.

### 2.2 Contrast-Enhanced Representation Learning

Recently, contrastive learning has become increasingly popular across a range of computer vision domains, including image representation learning,^38,39^ video representation learning,^40,41^ time series representation learning.^42,43^ Among these approaches, contrastive loss^15,38,39^ and triplet loss^44,43^ have emerged as two prevalent loss functions for conducting contrasting tasks. The foundational concept underlying the former is to bring together representations of augmented samples from the same image or video clips (i.e., positive pairs), while simultaneously pushing apart those originating from different instances (i.e., negative pairs). The latter shares the same objective but involves defining a triplet consisting of anchor, positive, and negative pairs. The positive pairs composed of anchor and positive representations are encouraged to be close, whereas the negative pairs comprising anchor and negative representations should be far away.

Different from,^15^ our contrastive learning strategy is built on the triplet loss where we explicitly construct the anchor, positive and negative representations based on the sequence representation **X** and structure representation **G** for enhancing representation learning. In particular, we utilize prior information on the amino acid labels to guide the construction of positive and negative pairs. Specifically, we first sample a fixed number of amino acids from **X** and **G** to form **X**_**s**_ and **G**_**s**_ (In experiments, we sample *L*_*s*_ amino acids in each mini-batch). This is because it is too computationally expensive to measure each amino acid in a mini-batch. Then we identify **G**_*s*_ and **X**_*s*_ as positive and anchor representations, respectively, and our goal is to pull them (i.e., positive pairs) together. Inspired by,^43^ given **G**_*s*_ and **X**_*s*_, we formalize the objective function of similarity learning between amino acids as
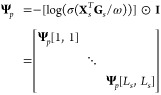
3where ⊙ represents the element-wise product. **I** ∈  represents the identity matrix used to select pairwise amino acids with the same index, and 0 < ω < 1 is a scale factor to adjust the correlation between vectors. **Ψ**[, ] is the element of matrix **Ψ**.

Afterward, we can obtain the overall loss  between positive pairs below:
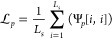
4

In eq 3, we construct positive pairs based on the inherent similarity between sequence and structure representations for direct contrast. The representations of pairwise amino acids with the same index in **G**_*s*_ and **X**_*s*_ indicate the same amino acid. In contrast, the construction of negative pairs relies on prior amino acid labels. In detail, for each amino acid in anchor representation **X**_*s*_, we sample *K* amino acids that have different labels to it from **X**_*s*_ to form the set of negative representations, i.e., . The element  can be represented as  where *l*[*j*] is the class label of the *j*-th amino acid residue. Therefore, the negative representation  and the anchor representation **X**_*s*_ should be separate. The function  to be minimized with the corresponding loss  is expressed as
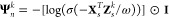
5
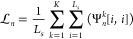
6Afterward, we can obtain the contrastive loss  as follows:
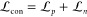
7

### 2.3 Structure-Sequence Feature Aggregation

In the field of protein analysis, it is crucial to acknowledge the inherent diversity of protein lengths. Proteins can range from relatively short sequences to incredibly long ones, and this variation poses a challenge when aiming to capture their overall characteristics. To effectively characterize a protein, we need to aggregate the distinctive features of all its constituent amino acids into a unified protein-level representation.^45^ This aggregation process allows us to encapsulate the essential information contained within the entire protein sequence, enabling a more comprehensive and meaningful analysis of its structural and functional attributes. Similar to previous studies,^46,47^ we employ self-attention pooling to aggregate all amino acid features. Given the structure representation **G**, the attention matrix  can be computed as
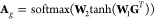
8where  and  are two learnable parameters. *D^″^* is the number of groups of attention vectors, each of which evaluates the association of each residue with thermostability from a specific perspective. The softmax(·) is performed along the second dimension of its input. We then compute the aggregated protein embedding **G̃** by multiplying the attention matrix **A**_*g*_ and the encoded structure representation **G**, and average all groups of attention vectors as follows:
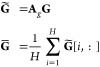
9where **G̃**[*i*,:] denotes the *i*-th row of the matrix **G̃**.  is the final graph-level representation. Similarly, we apply the same self-attention pooling to sequence representation **X** to obtain the final representation . Finally, we combine the two types of representations (i.e.,  and ) by the concatenation operation, followed by adopting a fully connected layer to generate prediction results. By combining the traditional prediction loss (cross-entropy loss for classification and root-mean-square error (RMSE) for regression) with our proposed contrastive loss, we are able to produce more accurate results. The overall loss is defined as
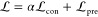
10where α is the weight of .

## 3 Experimental Setup

### 3.1 Data Sets

HotProtein data set is a large-scale protein data set with organism-level temperature annotations for both classification and regression tasks.^15^ The detailed experimental protocol for obtaining the values of thermostability can be found in ref (14). Briefly, the thermostability is measured as a lower bound of the protein’s melting temperature, which can be used for regression prediction. It contains 182 K amino acid sequences of proteins from 230 different species, covering five thermostability types, e.g., Cryophilic (−20–5 Celsius), Psychrophilic (5–25 Celsius), Mesophilic (25–45 Celsius), Thermophilic (45–75 Celsius), and Hyperthermophilic (>75 Celsius). HotProtein consists of 4 distinct subsets with different scales, including HP-S^2^C2 (2 classes), HP-S^2^C5 (5 classes), HP-S (5 classes) and HP-SC2 (2 classes) as developed in ref (15). Briefly, four distinct testbeds are derived from the HotProtein data set that differed in scale: (1) HP-S^2^C2 has 1026 “hot” (≥45 °C) and 939 “cold” (<45 °C) proteins from 61 and 4 species, respectively. (2) HP-S^2^C5 consists {73, 387, 195, 196, 189} proteins sampled from the five categories, from Cryophilic to Hyperthermophilic. (3) HP-S is the entire sequence HotProtein data set with {6390, 34946, 30333, 79087, 31549} sequences from {3, 32, 31, 116, 48} different species, of five classes ordered from Cryophilic to Hyperthermophilic. (4) HP-SC2 is a 2-class variant created by merging Hyperthermophilic and Thermophilic as “hot” class and the other three as “cold” class. Given their sample size, HP-S^2^C2/C5 and HP-S/SC2 are regarded as small- and large-scale data sets.

The second data set used in this work is another large-scale protein thermostability data set developed in DeepStabP,^48^ derived from the Meltome Atlas.^14^ It contains 35,112 protein sequences retrieved from UniProt^49^ with annotated melting temperatures from diverse species.

### 3.2 Evaluation Metrics

The performance of the methods in classification is evaluated with two measures as fellows:
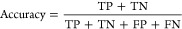
11
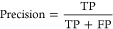
12Accuracy measures the overall correctness of the model across the entire data set. Precision evaluates a model’s classification performance on a specific class, which represents the proportion of samples predicted as the positive class that are true positives. TP represents True Positives (correctly predicted hot proteins), TN represents True Negatives (correctly predicted cold proteins), FP represents False Positives (cold proteins incorrectly predicted as hot), and FN represents False Negatives (hot proteins incorrectly predicted as cold).

The Area under the receiver operating characteristic curve (AUC-ROC or AUC) evaluates the model’s ability to distinguish between classes across various classification thresholds. The ROC curve plots the True Positive Rate (TPR) against the False Positive Rate (FPR) at different threshold values, where
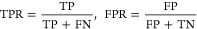
13The AUC ranges from 0 to 1, with 1 representing perfect classification and 0.5 indicating a random guess. A higher AUC reflects better model discrimination between classes of melting temperatures across different decision thresholds.

We evaluate the regression performance using three metrics: Pearson correlation coefficient, Spearman correlation coefficient, and *R*^2^. The Pearson correlation coefficient measures the strength and direction of the linear relationship between two continuous variables. It quantifies this relationship by computing the covariance between the two variables divided by the product of their respective standard deviations, where its values range from −1 (perfect negative correlation) to 1 (perfect positive correlation). The formula for the Pearson correlation coefficient is defined as
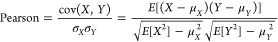
14where cov(·) is the covariance. σ_*X*_ and σ_*Y*_ are the standard deviations of *X* and *Y* (ground-truth and predicted values), respectively. μ_*X*_ and μ_*Y*_ are the mean values of *X* and *Y*. *E* is the expectation.

The Spearman correlation coefficient assesses the strength and direction of monotonic relationships between two variables. It achieves this by first converting the observed values of each variable into ranks and then calculating the Pearson correlation coefficient between these ranks. The formula for the Spearman correlation coefficient is as follows:
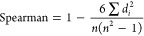
15where *d*_*i*_ represents the differences between ranks for each pair of observations. *n* is the sample size.

*R*^2^ measures how well the model fits the data. It represents the proportion of variance in the dependent variable explained by the independent variables. *R*^2^ ranges from 0 to 1, where 1 indicates a perfect prediction. The formula is
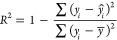
16where *y*_*i*_ are the actual values, *ŷ*_*i*_ are the predicted values, and *y̅* is the mean of actual values.

### 3.3 Implementation Details

All models were trained on a single NVIDIA A100 40GB GPU using PyTorch. We employed the Adam optimizer with fixed learning rates throughout the training. All models were trained for 10 epochs, and we report the test set performance of the best-performing model selected across epochs. For model training, we used a batch size of 4 for HP-S^2^C2 and HP-S^2^C5, 8 for DeepStabP, and 512 for HP-S. The learning rates were set to 8e-5 for HP-S^2^C2, 8e-4 for HP-S^2^C5, and 1e-4 for HP-S and DeepStabP.

## 4 Results

Using the large-scale HotProtein data set, we evaluated the performance of two configurations of models: ProCeSa, which purely integrates structural and sequential information, and ProCeSa(+Cont), which additionally performs contrastive learning of the two protein representations. The results show that our proposed ProCeSa has a better performance compared to those of other SOTA models.

We compare ProCeSa to five outstanding models: 3D GCN,^50^ TAPE,^51^ ESM-IF1,^35^ ESM-1B,^29^ and HotProtein.^15^ For a fair comparison, we use the same data sets as in HotProtein.^15^ The results of the 3D GCN, TAPE, ESM-IF1, ESM-1B, and HotProtein model are cited from the original HotProtein study.^15^ We also compare the performance of ProCeSa installed with different encoders of ESM-1B and ESM-C.^52^

For classification tasks, accuracy is utilized as the primary evaluation metric for both two-class and five-class classification tasks, while binary precision is specifically calculated for the two-class classification scenario (Table 1). Across all subdata sets of the HotProtein benchmark, ProSeCa consistently outperforms all baseline models. Furthermore, by leveraging the advanced ESM-C encoder in conjunction with contrastive learning, ProSeCa demonstrates superior performance in the majority of cases.

**Table 1:** Performance of Predicting Thermostability with Classification Tasks Using the HotProtein Dataset; Accuracy (%) Is Reported for All Three Datasets, and Precision (%) Is Calculated for the Two-Class Classification on HP-S^2^C2 and HP-SC2

	HP-S^2^C2		HP-S^2^C5	HP-S	HP-SC2	
method	accuracy	precision	accuracy	accuracy	accuracy	precision
3D GCN^50^	78.88 ± 1.57	73.39 ± 2.76	67.40 ± 2.11	N/A	N/A	N/A
TAPE^51^	83.31 ± 1.10	76.42 ± 3.06	66.44 ± 2.30	64.75 ± 0.23	76.37 ± 0.25	80.64 ± 0.50
ESM-IF1^35^	79.08 ± 0.85	76.49 ± 3.96	58.75 ± 2.46	N/A	N/A	N/A
ESM-1B^29^	91.19 ± 0.47	84.18 ± 1.71	83.26 ± 1.54	69.50 ± 0.16	86.24 ± 0.22	88.14 ± 1.62
HotProtein^15^	92.36 ± 0.58	86.51 ± 1.67	86.25 ± 1.03	73.21 ± 0.13	87.57 ± 0.10	89.07 ± 1.29
ProCeSa-ESM-1B	92.94 ± 0.53	89.30 ± 2.83	87.02 ± 1.88	74.54 ± 0.02	87.87 ± 0.15	91.20 ± 0.48
ProCeSa-ESM-1B (+Cont)	93.39 ± 1.07	**91.38** ± **2.68**	87.50 ± 2.31	75.52 ± 0.10	88.13 ± 0.28	91.38 ± 0.43
ProCeSa-ESM-C	93.96 ± 0.34	89.57 ± 0.91	92.85 ± 0.06	80.37 ± 0.06	91.15 ± 0.16	**92.97** ± **1.38**
ProCeSa-ESM-C (+Cont)	**94.17** ± **0.06**	89.52 ± 0.40	**93.36** ± **0.45**	**80.53** ± **0.31**	**91.15** ± **0.14**	92.48 ± 1.72

For regression tasks, the model performance is evaluated using Spearman and Pearson correlation coefficients. Analyzing different subsets of the HotProtein data set (Table 2), ProSeCa equipped with the ESM-C encoder and contrastive learning achieves the best performance. These results highlight the effectiveness of the ESM-C encoder in enhancing the representation quality. Additionally, contrastive learning contributes to the improved modeling of both sequence and structural features, although the observed performance gains are relatively modest.

**Table 2:** Performance of Predicting Thermostability with Regression Tasks Using the HotProtein Dataset^a^

	HP-S^2^C2		HP-S^2^C5		HP-S	
method	Spearman	Pearson	Spearman	Pearson	Spearman	Pearson
3D GCN^50^	0.490 ± 0.019	0.469 ± 0.019	0.291 ± 0.053	0.301 ± 0.074	N/A	N/A
TAPE^51^	0.432 ± 0.061	0.386 ± 0.065	0.367 ± 0.063	0.364 ± 0.047	0.504 ± 0.013	0.453 ± 0.031
ESM-IF1^35^	0.589 ± 0.040	0.547 ± 0.036	0.373 ± 0.036	0.377 ± 0.035	N/A	N/A
ESM-1B^29^	0.890 ± 0.018	0.893 ± 0.024	0.712 ± 0.043	0.804 ± 0.023	0.807 ± 0.001	0.809 ± 0.001
HotProtein^15^	0.906 ± 0.010	0.923 ± 0.012	0.754 ± 0.035	0.837 ± 0.019	0.823 ± 0.001	0.827 ± 0.003
ProCeSa-ESM-1B	0.930 ± 0.012	0.958 ± 0.013	0.856 ± 0.053	0.900 ± 0.039	0.852 ± 0	0.858 ± 0
ProCeSa-ESM-1B (+Cont)	0.933 ± 0.014	0.961 ± 0.015	0.861 ± 0.050	0.904 ± 0.045	0.855 ± 0.001	0.861 ± 0.001
ProCeSa-ESM-C	0.942 ± 0.001	0.969 ± 0.000	0.919 ± 0.000	0.949 ± 0.002	0.892 ± 0.000	0.897 ± 0.001
ProCeSa-ESM-C (+Cont)	**0.943** ± **0.001**	**0.969** ± **0.000**	**0.921** ± **0.002**	**0.954** ± **0.005**	**0.894** ± **0.001**	**0.900** ± **0.001**

a Correlation coefficients of Spearman and Pearson are reported for all three datasets; 95(%) confidence intervals are computed via the 10-fold evaluation on HP-S^2^C2/C5 and 3 replicates on HP-S.

To evaluate the generalizability of the ProCeSa model, we tested its performance on another thermostability data set, DeepStabP^48^ for the regression task. Spearman correlation, Pearson correlation, and *R*^2^ values were calculated to ensure a fair comparison with the results reported in the original DeepStabP study^48^ (Table 3). The ProCeSa-ESM-C model shows comparable performance compared to the DeepStabP model based on the ProtT5-XL PLM. Notably, ProCeSa achieves this level of performance with significantly fewer parameters (600 M vs 3B), highlighting its efficiency in capturing capabilities comparable to those of larger models.

**Table 3:** Performance of Predicting Thermostability with Regression Tasks Using the Dataset in DeepStabP^a^

method	*R*^2^	Spearman	Pearson	model parameter
DeepStabP (ProtT5-XL)^48^ (reproduced)	0.792 ± 0.021	0.732 ± 0.005	0.897 ± 0.003	3B
ProCeSa-ESM-C (+Cont)	0.788 ± 0.004	0.717 ± 0.004	0.889 ± 0.004	600M

a *R*^2^, Spearman, and Pearson correlation coefficients are reported. The approximate number of model parameters is listed for both models.

HotProtein makes use of AlphaFold2 to predict protein 3D coordinates and feeds this 3D information to their model. Considering taking advantage of interactions between amino acids, we extract the graph information using ESM-series models following HybridGCN^53^ and feed the extracted contact map into the GCN network. This allows our model to fully learn the interactions between amino acids, thereby improving the accuracy of predictions and the generalization ability of the model. On the other hand, contrastive learning can bring an increase in performance. These stable improvements demonstrate that our specially designed triplet-loss contrastive learning mode for two different modalities enables the model to explore the similarities and differences between the protein structure and sequence.

**Figure 2:**
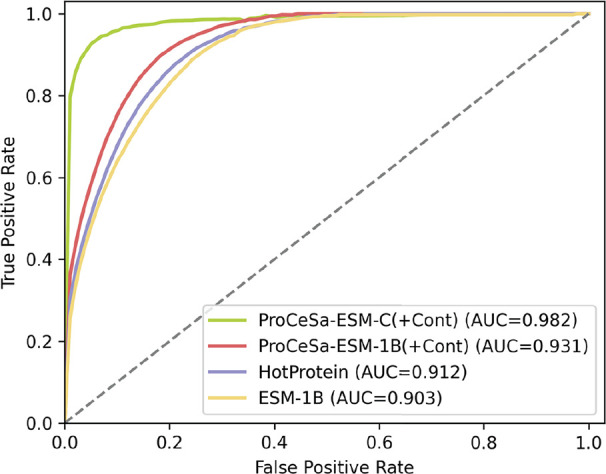
ROC curves performance comparison of ESM-1B, HotProtein, ProCeSa-ESM-1B, and ProCeSa-ESM-C models on HP-S^2^C5.

We compare the macro ROC curves of models on the HP-S^2^C5 subdata set (Figure 2). Macro ROC is selected over micro ROC because it evaluates the performance of each class equally, which is important given the class imbalance in this data set. Our ProCeSa(+Cont) demonstrates superior performance compared with the other models. The performance difference between ProCeSa-ESM-C and ProCeSa-ESM-1B indicates that ESM-C extracts features more effectively than ESM-1B, leading to better prediction accuracy.

**Figure 3:**
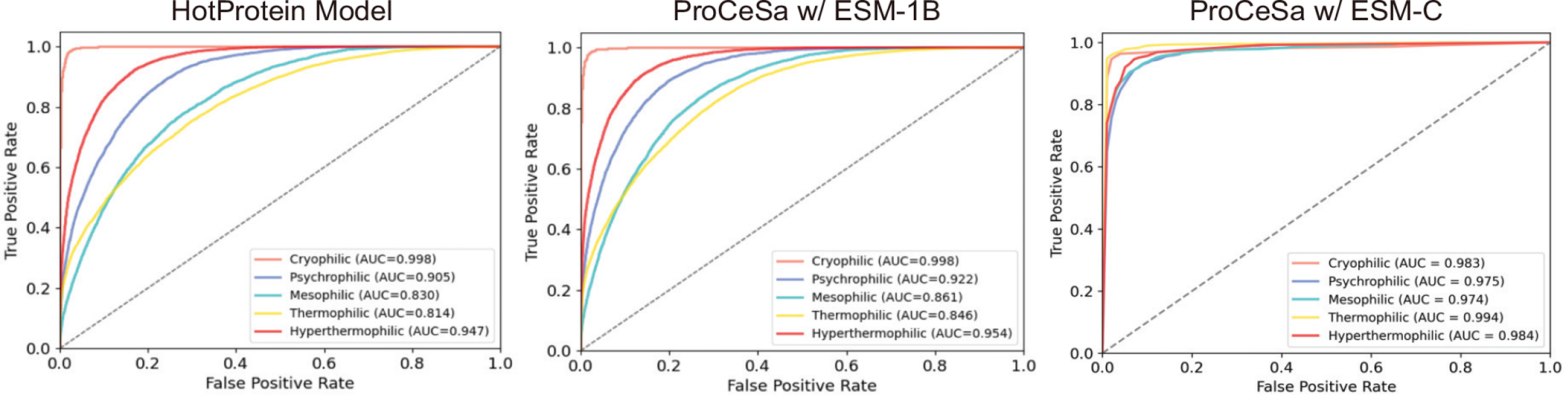
Individual ROC curves comparison of HotProtein, Procesa-ESM-1B, and Procesa-ESM-C on 5 classes of the HP-S^2^C5 Data set.

Using macro ROC curves to evaluate performance across the five protein categories in HP-S^2^C5 (Figure 3), we observe distinct trends. Most models achieve their highest AUC in the Cryophilic category with scores ranging from 0.997 to 0.998. However, ProCeSa-ESM-C achieves its highest AUC in the thermophilic category (0.994). This difference may stem from ProCeSa-ESM-C being pretrained on data that better captures features relevant to high-temperature proteins, allowing it to perform particularly well in the thermophilic category.

In the mesophilic category, ESM-1B and ProCeSa-ESM-C show the lowest AUC values (0.800 and 0.974, respectively). HotProtein and ProCeSa-ESM-1B perform the worst in the Thermophilic category, with AUC scores of 0.814 and 0.846, respectively. Notably, ProCeSa-ESM-C demonstrates a balanced performance across all categories, suggesting its robustness in handling diverse protein types.

These findings highlight the importance of considering the specific protein category when training thermostability prediction models. Future work could focus on improving performance in individual categories.

## 5 Discussion

In this work, we introduce ProCeSa, a novel deep-learning network model tailored specifically for predicting protein thermostability. ProCeSa uniquely leverages structural and sequential features extracted by large language models, pioneering a departure from conventional approaches that rely solely on one type of feature or on conventional biophysical features. By the integration of both types of features, our model capitalizes on the complementary nature of structural and sequential information, thus enriching the predictive capacity for protein thermostability.

Central to ProCeSa is the utilization of a contrastive learning scheme, which enables the exploration of implicit interactions between structural and sequence features. This approach not only enhances the model’s ability to capture meaningful relationships within protein sequences and structures but also facilitates robust performance across diverse data sets.

Contrastive learning has been recently used for various tasks in protein engineering.^54−56^ The primary benefit of this learning scheme is to reduce dependence on labeled data. In protein sequence analysis, labeled data can be scarce. Contrastive learning effectively utilizes unlabeled data, mitigating the need for extensive labeled data sets and facilitating the development of accurate models even in data-constrained scenarios, particularly for enzyme functional annotation, such as enzyme commission (EC) number.^57^ Beyond data efficiency, contrastive learning enhances model robustness through controlled noise during training, helping models learn stable protein representations and reduce overfitting.^56^ This approach also improves the discrimination ability between similar proteins, which is crucial for tasks like identifying true binding partners in drug-target interactions.^55^ Models trained with contrastive objectives show better generalization across diverse protein families and maintain consistent performance on previously unseen sequences.^54^

Compared to the abundant protein sequence databases, high-quality experimentally determined structural data remain scarce for most proteins. Recent advancements in structure prediction from sequences, such as RoseTTAFold,^58,59^ AlphaFold,^24,60^ ESMfold,^31^ and ESM Cambrian,^52^ have made it possible to generate 3D atomic structures from virtually any protein sequence. Incorporating these predicted structures into computational models has been shown to enhance performance in general-purpose protein engineering tasks, as demonstrated by models such as S-PLM^61^ and ProTrek,^62^ as well as in specific applications like thermostability prediction, exemplified by SPIRED^25^ and ProSTAGE.^26^

S-PLM, for instance, utilizes contact maps derived from physical distances in predicted 3D structures, while attention-based contact maps generated by ESM language models capture both physical contacts and functional relationships between residues, independent of the structure prediction accuracy. Predicted 3D structures have proven useful for estimating relative stability changes (ΔΔ*G* or Δ*T*_m_) between wild-type and mutated proteins.^22,23^ However, recent studies have highlighted that thermostability predictions are highly sensitive to structural precision, and even state-of-the-art structure prediction models cannot consistently deliver the level of precision required for reliable ΔΔ*G* calculations.^27^ This highlights the need to develop models capable of directly predicting melting temperatures (*T*_m_) from sequence information without relying on detailed structural features. Such models would significantly advance protein engineering by reducing dependence on expensive and time-consuming wet-lab experiments.^28^

Future advancements in thermostability prediction will depend on the integration of diverse methodologies, combining data from multiple levels of protein characterization.^63^ Both the HotProtein and DeepStabP data sets are derived from the experimental Meltome Atlas,^14^ which primarily reflects organismal optimal growth temperatures. While these temperatures show some correlation with protein thermostability, they are not always reliable proxies. Instead, they can be considered a lower bound for the melting temperature (*T*_m_), a parameter that is relatively easy to measure through wet-lab experiments. In practical applications, however, the most useful predictor of thermostability is the change in the free energy (ΔΔ*G*). Unfortunately, all deep learning models face limitations due to how databases are annotated. For example, critical factors such as pH and salinity, which significantly influence thermostability, are poorly represented in existing open-access data sets. Additionally, the sequences in HotProtein often lack full structural information, particularly in regions with large unstructured segments—a common issue across data sets used for training deep learning models. This poses a significant challenge when encoding detailed structural features, especially when relying on limited predictability accuracy from structure prediction models such as AlphaFold or other methods.^27^ Addressing these gaps will be essential for improving the accuracy and applicability of thermostability prediction models.

Existing classical thermostability prediction models have advanced by incorporating key structural- and sequence-based factors. PROSS^64,65^ is a computational framework designed to engineer stabilized protein variants without compromising their functional activity. The method begins by analyzing a high-resolution protein structure and multiple sequence alignments of homologous sequences. It then employs a series of steps of optimization to enhance protein stability by phylogenetic filtering for evolutionarily conserved amino acids and eliminating destabilized mutations to select the most stabilizing mutations while ensuring the protein’s functional integrity is preserved. PROSS achieves its goals by targeting specific regions of the protein: core residues are modified to improve packing efficiency, surface residues are adjusted to reduce aggregation and backbone interactions are optimized to enhance rigidity. As a result, PROSS-designed proteins often exhibit significantly increased thermostability and higher expression levels in bacterial systems. Additionally, these stabilized variants frequently fold correctly without the need for chaperone assistance, making them more suitable for industrial and biomedical applications. This approach demonstrates how computational design can effectively bridge the gap between protein stability and functionality.

With the recent advances in PLMs, a two-stage prediction framework could effectively address existing limitations by integrating the strengths of PLM-based models and classical models such as PROSS. In the first stage, a foundation PLM, such as the one proposed in this study, could provide an initial estimation of protein thermostability directly from sequence information. This would enable rapid screening of new protein sequences for potential thermostability, leveraging the metric of melting temperature. By predicting *T*_m_ values, the PLM could identify sequences with favorable stability profiles, serving as a high-throughput filter for further analysis. In the second stage, more detailed structural information could be integrated to refine predictions.^27^ This stage would involve the selection and elimination of single or multiple point mutations,^66^ capturing the nuanced sequence-structure–function relationships. PROSS could then be applied to optimize these mutations, focusing on core packing, surface residue adjustments, and backbone interactions to further enhance the stability and expression levels. This hierarchical approach would combine the speed and scalability of PLMs with the precision of structure-based methods such as PROSS, significantly improving predictive accuracy and applicability. By bridging sequence-based predictions with experimentally validated structural insights, this framework would not only enhance thermostability predictions but also accelerate advancements in protein engineering. It would enable researchers to design proteins with improved coarse-grained thermostability (*T*_m_) and fine-grained energetically favorable folding (ΔΔ*G*), reducing reliance on experimental screening and facilitating the development of proteins for industrial, therapeutic, and biotechnological applications.

## Data Availability

The code and data used to generate the results in this work are available on the project page: https://github.com/notabigfish/procesa.

## Supporting Information Available

Replies to the comments from the editor and referee (PDF)
