# Supplementary material for: ProCeSa: Contrast-Enhanced Structure-Aware Network for Thermostability Prediction with Protein Language Models
Source: J Chem Inf Model. 2025 Feb 24;65(5):2304–13. doi: 10.1021/acs.jcim.4c01752 (PMC11898056; doi:10.1021/acs.jcim.4c01752)
Supplement: Supplementary file 1 — ci4c01752_si_001.pdf [file ci4c01752_si_001.pdf]

Journal of Chemical Information and Modeling (ci-2024-01752x.R1)

Dear Editor and Reviewers:

First of all, we would like to express our gratitude to you for carefully reading our manuscript and giving us constructive suggestions. We have carefully considered your comments and revised our manuscript in light of your remarks and suggestions. You are generous in sharing your time for carefully reading our manuscript and your expertise in facilitating our work. Thank you all for your comments during the revision of our manuscript.

The following are point-to-point responses to the reviewers' comments. The reply is arranged in Q's and A's. We first quote your comments, and then give our answers after each of your questions.

Replies to the Comments from Editor:

a) A point-by-point response to the reviewers.
b) A clean, unmarked copy of the manuscript document file.
c) A marked copy of the revised manuscript in which all the changes are clearly tracked or highlighted. Upload this file as Supporting Information for Review.
d) A clean, unmarked Supporting Information for Publication file, without any highlighting or tracked changes.
Answer: We have carefully revised our manuscript in light of the comments provided by the reviewer. We provided a version of the revised manuscript with highlights denoting where the text has been revised (colored in red).

Replies to the Comments from Referee#1:

After carefully reviewing the authors' revisions, I confirm that they have addressed all of my comments and concerns to my satisfaction. Therefore, I am pleased to recommend that the paper is now ready for publication.
Thanks for the endorsement.

Replies to the Comments from Referee#3:

The manuscript by Liu and coworkers presents a new method for predicting protein thermostability, called ProCeSa, a model that integrates both sequence and structural information using a contrastive learning framework. By leveraging amino acid residue categories, the model aims to identify complex structural patterns relevant to thermostability. This referee is not an expert in protein language models; my expertise lies more in the use of structure-based classical methods for predicting thermostability. Therefore, please consider my judgment with caution.
In any case, I concur with the other reviewers that this method may have limited utility for real-life applications, given that the training dataset reflects optimal growth conditions rather than sequence- and structure-dependent thermostability.
The ultimate predictor of thermostability is ΔΔG (not so much Tm, although the latter is easier to obtain), and virtually all methods suffer from the same deficiencies due to how databases are annotated. For instance, pH and salinity are major determinants of thermostability, yet the HotProtein dataset does not capture this critical information. Moreover, it is unclear whether the sequences in HotProtein correspond exclusively to fully structured proteins or if they also include large unstructured

regions, which is common when analyzing full-length genomic sequences. This could pose a significant issue when encoding structural features with low predictability values derived from blind AlphaFold predictions or other structure prediction methods.
I also agree that other robust and widely used thermostability prediction methods, particularly PROSS, should be tested and compared to ProCeSa.

A: Thank you for your insightful suggestion from an experimentalist's perspective. We agree with the Reviewer that significant gaps remain between current AI models and real-life applications, primarily due to incomplete experimental features in existing datasets and the limited structural predictability of AI models, even using AlphaFold, as mentioned by the Reviewer.

The PROSS is a valuable tool, while it is difficult to apply it to the HotProtein data directly, as the data are largely not fully structured. We have added discussion about the PROSS model. Currently, it is very challenging to directly predict $\Delta\Delta G$ using AI-based protein language (PLM) models. While most PLMs offer coarse-grained predictions, such as melting temperature (Tm) values, methods like PROSS provide fine-grained predictions, including changes in free energy ($\Delta\Delta G$). To bridge this gap for real-life applications, a combined approach could be employed: leveraging PLMs for large-scale, full-sequence screening and using tools like PROSS for fine-tuning single or multiple point mutations.

We are actively addressing these challenges by constructing more comprehensive datasets and developing two-stage modeling frameworks that integrate both coarse- and fine-grained predictions. To better reflect these considerations and our ongoing efforts, we have revised the manuscript to include additional discussion, including discussion on PROSS, on this topic (highlighted in red text at the end of the manuscript).

Below is a snapshot of the text highlighted in red for easy review.

Future advancements in thermostability prediction will depend on the integration of diverse methodologies, combining data from multiple levels of protein characterization [63]. Both the HotProtein and DeepStabP datasets are derived from the experimental Meltome Atlas [14], which primarily reflects organismal optimal growth temperatures. While these temperatures show some correlation with protein thermostability, they are not always reliable proxies. Instead, they can be considered a lower bound for the melting temperature (T_m), a parameter that is relatively easy to measure through wet-lab experiments. In practical applications, however, the most useful predictor of thermostability is the change in free energy ($\Delta\Delta G$). Unfortunately, all deep learning models face limitations due to how databases are annotated. For example, critical factors such as pH and salinity, which significantly influence thermostability, are poorly represented in existing open-access datasets. Additionally, the sequences in HotProtein often lack full structural information, particularly in regions with large unstructured segments—a common issue across datasets used for training deep learning models. This poses a significant challenge when encoding detailed structural features, especially when relying on limited predictability accuracy from structure prediction models such as AlphaFold or other methods [27]. Addressing these gaps will be essential for improving the accuracy and applicability of thermostability

prediction models.

Existing classical thermostability prediction models have advanced by incorporating key structural and sequence-based factors. PROSS [64], [65] is a computational framework designed to engineer stabilized protein variants without compromising their functional activity. The method begins by analyzing a high-resolution protein structure and multiple sequence alignment of homologous sequences. It then employs a series of steps of optimization to enhance protein stability by phylogenetic filtering for evolutionarily conserved amino acids and eliminating destabilized mutations to select the most stabilizing mutations while ensuring the protein's functional integrity is preserved. PROSS achieves its goals by targeting specific regions of the protein: core residues are modified to improve packing efficiency, surface residues are adjusted to reduce aggregation, and backbone interactions are optimized to enhance rigidity. As a result, PROSS-designed proteins often exhibit significantly increased thermostability and higher expression levels in bacterial systems. Additionally, these stabilized variants frequently fold correctly without the need for chaperone assistance, making them more suitable for industrial and biomedical applications. This approach demonstrates how computational design can effectively bridge the gap between protein stability and functionality.

With the recent advances in PLMs, a two-stage prediction framework could effectively address existing limitations by integrating the strengths of PLM-based models and classical models such as PROSS. In the first stage, a foundation PLM, such as the one proposed in this study, could provide an initial estimation of protein thermostability directly from sequence information. This would enable rapid screening of new protein sequences for potential thermostability, leveraging the metric of the melting temperature. By predicting T_m values, the PLM could identify sequences with favorable stability profiles, serving as a high-throughput filter for further analysis. In the second stage, more detailed structural information could be integrated to refine predictions [27]. This stage would involve the selection and elimination of single or multiple point mutations [66], capturing the nuanced sequence-structure-function relationships. PROSS could then be applied to optimize these mutations, focusing on core packing, surface residue adjustments, and backbone interactions to further enhance stability and expression levels. This hierarchical approach would combine the speed and scalability of PLMs with the precision of structure-based methods like PROSS, significantly improving predictive accuracy and applicability. By bridging sequence-based predictions with experimentally validated structural insights, this framework would not only enhance thermostability predictions but also accelerate advancements in protein engineering.

It would enable researchers to design proteins with improved coarse-grained thermostability (T_m) and fine-grained energetically favorable folding ($\Delta\Delta G$), reducing reliance on experimental screening and facilitating the development of proteins for industrial, therapeutic, and biotechnological applications.

On a more practical note, I was unable to use the GitHub code to predict the Tm of a set of approximately 50 proteins whose Tm we measured in our lab. As a result, I do not find the model particularly useful at this stage, at least for non-experts.

A: Thank you for reviewing and testing our code. When working with more constrained datasets, such as those limited to 50 proteins, it is essential to account for specific factors tailored to small-scale data, similar to considerations in other AI models. We appreciate the feedback and are open to collaborating with the Reviewer to explore potential applications to such data, should the opportunity arise.

Finally, the supporting information appears to be merely a copy of the main article; I could not find correlation graphs or information about the datasets there.

A: Sorry for the confusion. The supporting information provided in the system is only a requirement of submission, with both high-lighted and clear versions of the manuscript. Therefore, no additional information is listed in the supporting information.
